# Comparison of Hospital Readmission and Mortality between COVID-19 and Pneumonia Patients

**DOI:** 10.3390/jcm11144199

**Published:** 2022-07-20

**Authors:** Mesnad Alyabsi, Omar Aldibasi, Mohammad Bosaeed, Maha Alanazi, Anwar Alqarni, Bayan Albdah, Naif Khalaf Alharbi, Suliman Alghnam

**Affiliations:** 1 Population Health Research Section, King Abdullah International Medical Research Center, P.O. Box 22490, Riyadh 11426, Saudi Arabia; anwarralqarni@gmail.com (A.A.); ghnams@ngha.med.sa (S.A.); 2 King Saud Bin Abdulaziz University for Health Sciences, P.O. Box 22490, Riyadh 11426, Saudi Arabia; aldibasiom@ngha.med.sa (O.A.); bosaeedmo@ngha.med.sa (M.B.); alanazima32@ngha.med.sa (M.A.); albdahba@ngha.med.sa (B.A.); harbina2@ngha.med.sa (N.K.A.); 3 Section of Biostatistics, King Abdullah International Medical Research Center, P.O. Box 22490, Riyadh 11426, Saudi Arabia; 4 King Abdullah International Medical Research Center, P.O. Box 22490, Riyadh 11426, Saudi Arabia; 5 Department of Medicine, King Abdulaziz Medical City, Ministry of National Guard Health Affairs, Riyadh 11426, Saudi Arabia; 6 Health and Rehabilitation Sciences College, Princess Noura Bint Abdul Rahman University, Riyadh 11564, Saudi Arabia

**Keywords:** Saudi Arabia, COVID-19, patient readmission, pneumonia

## Abstract

Coronavirus disease 2019 (COVID-19) survivors can have lasting signs and symptoms, including various organ damage, indicating that COVID-19 can be a chronic illness. The current study aims to compare the 30-day hospital readmission and death rate of patients admitted to the hospital with COVID-19 and pneumonia due to other causes. A retrospective cohort study was conducted using data from the Saudi National Guard Health Affairs (NGHA). Records of patients admitted with COVID-19 between 1 March 202 and 31 December 2020 (*n* = 3597) and pneumonia during 2017 and 2019 (*n* = 6324) were retrieved and analyzed. We compared the likelihood of 30-day hospital readmission, intensive care unit (ICU) admission, and death between the two groups. Compared with the control group, COVID-19 patients had higher odds of 30-day readmission (odds ratio 1.90, 95% confidence interval 1.61–2.24), higher risk of ICU transfer (hazard ratio 1.85, 95% confidence interval 1.65–2.07), more extended hospital stay (7 vs. 4 days), but less risk of death (hazard ratio 0.18, 95% confidence interval 0.14–0.24). The findings that hospital readmission was higher in COVID-19 recovered patients than in other pneumonia patients inform the current discussion about readmission and death in COVID-19 patients.

## 1. Introduction

The Coronavirus Disease 2019 (COVID-19) caused by SARS-CoV-2 infection has led to a major pandemic that resulted in over four hundred million confirmed cases and more than 5.9 million deaths worldwide, as of 28 February 2022 [1]. Initially described as an acute pulmonary illness, COVID-19 turned out to be a systemic disease that can progress to the dysfunction of multiple organ systems and death, particularly in vulnerable populations such as those with comorbid conditions. Rapidly growing evidence revealed that many survivors of COVID-19 can sustain persistent signs and symptoms, and multiple organ injuries after the infection has subsided, suggesting that COVID-19 can also be a chronic condition [2]. 

After being infected with COVID-19, people can experience post-recovery conditions for up to four weeks. This represents a wide range of new, returning, or ongoing health problems, including persistent symptoms such as anosmia, breathing difficulty, fatigue, and emotional instabilities [3,4]. More importantly, many patients displayed new or worsening conditions such as heart failure, respiratory distress requiring invasive and non-invasive ventilation, neuropsychiatric disturbances resulting in readmission, and even death. For instance, the 30-day hospital readmission rates range from 4.5 to 11%, and the 60-day rates range between 20 and 21% [5,6,7,8,9,10,11,12,13,14,15,16]. Nonetheless, the magnitude of the long-term complications, the clinical and potentially genetic risk factors, and the mechanisms remain as of yet not fully elucidated. 

As of 28 February 2022, more than 744,000 cases were reported in Saudi Arabia, resulting in 8996 deaths [17,18]. Most of the published reports described the clinical outcome and the health service utilization among hospitalized patients with COVID-19 in Saudi hospitals [18,19,20,21,22,23,24,25,26,27,28,29,30]. So far, none have examined the rate and causes of hospital readmission, except a single study reported a rate of 9.6% of hospital readmission among children with COVID-19 [31]. In contrast, many international studies have shown that potential causes of readmission include hypoxic respiratory failure and thromboembolism. Moreover, they have identified that old-age (over 65), women, patients diagnosed with pneumonia, sepsis, and comorbidities such as hypertension, malignancy, heart failure, and renal diseases are the main predictors of readmission [5,6,7,8,9,10,11]. 

Therefore, the main objective of the current study was to estimate the rate and causes of 30-day readmission, together with risk factors in a large cohort of patients admitted with COVID-19 to five tertiary hospitals covering most of the regions of Saudi Arabia. A retrospective cohort of patients admitted with pneumonia served as a control group. This study aims to improving healthcare delivery, particularly planning long-term care for this category of patients. 

## 2. Materials and Methods

### 2.1. Data Sources 

In this observational retrospective cohort study, data were retrieved from the electronic health records at MNG-HA (BESTCare 2.0). The data have been described in previous publications [31,32,33]. In brief, the data cover more than 700,000 members receiving treatment at hospitals in five different regions of Saudi Arabia (Riyadh, Dammam, Alhasa, Madinah, and Jeddah). The data are electronic, and physicians enter the data directly into the system without any paper-based entry. In addition to patients’ demographic data, the electronic records capture outpatient and inpatient records, including the admission and discharge date. All extracted data were coded and analyzed anonymously. The Institutional Review Board of King Abdullah International Medical Research Center approved this study (IRB#RC20/358/R).

### 2.2. Study Population

**Figure 1:**
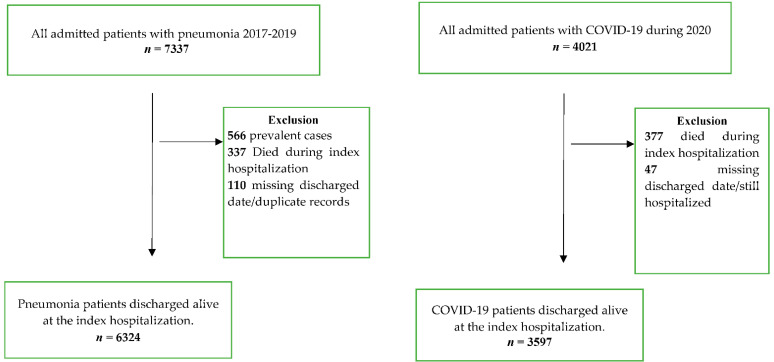
Flow diagram of the study population.

Participants included in the study were all eligible COVID-19 and pneumonia diagnosed patients who were registered in the MNG-HA hospitals system. There were 4021 patients admitted with COVID-19 during the year 2020. Among them, 377 patients died during index hospitalization while 47 patients were excluded for missing discharge date. Consequently, 3597 COVID-19 patients were involved in the final analysis. The control group consisted of 7337 individuals diagnosed with pneumonia between the years 2017 and 2019. Of 7337 eligible patients, 566 prevalent cases were excluded because our focus is on incident cases with first hospital admission. In addition to the 337 patients who died during the index hospitalization, 110 cases had missing discharge dates. Thus, 6324 pneumonia patients were included in the analysis (Figure 1).

### 2.3. Study Variables

In this study, several demographic and clinical characteristics were obtained, including patients’ age, gender, vital status, and comorbidities. Comorbidities included any previous diagnosis of hypertension (HTN), bronchial asthma, rheumatoid arthritis, cancer, depression, diabetes, dyslipidemia, and stroke. The number of comorbid conditions, length of hospital stay (LOS), and whether the patient had been transferred to the intensive care unit (ICU) were collected as well. In addition to the readmission, reasons for 30-day hospital readmission were reported and classified based on the affected body organ system.

### 2.4. Outcome Variables

Hospital readmission was defined as any hospitalization occurring within 30 days of discharge [5]. We obtained patients’ hospital readmission data within 30 days using the inpatient records based on the admission and discharge dates.

### 2.5. Statistical Analysis

We represented quantitative variables as mean and standard deviation or median and interquartile range for skewed variables. We expressed the qualitative variables as frequencies. We compared COVID-19 and pneumonia groups using Pearson χ^2^ tests, independent *t*-tests, or Wilcoxon tests. We conducted a univariate analysis to assess the relationship between each independent variable and patients’ readmission. Proportional hazard regression models were applied to model the outcome variables, including time to ICU transfer, readmission, and death. Results were reported in terms of hazard ratios. We used backward elimination in the multivariate analysis to exclude non-significant variables (*p* > 0.05), with the final model adjusting for the admitted cohort group, age, cancer, stroke, the number of comorbid conditions, and ICU admission.

We conducted sensitivity analyses to assess the robustness of our findings. We assessed the risk of 30-day hospital readmission after removing readmission due to planned/irrelevant admission. We also compared mortality during index hospitalization with mortality during the hospital readmission period. 

All statistical tests were 2-sided, and we considered the findings statistically significant at *p* < 0.05. We carried out all analyses using SAS statistical software version 9.4 and JMP version 16.1 (SAS Institute Inc., Cary, NC, USA). 

## 3. Results

### 3.1. Patients’ Characteristics 

The eligibility criteria are shown in Figure 1. A total of 110 patients were excluded because they had a missing discharge date, which is necessary to compute the rate of readmission. We included 9921 patients, of whom 3597 (36.2%) had COVID-19 and 6324 (63.7%) were pneumonia patients, respectively (Table 1). The average age was 50 years and half of the sample were males. Over 13.6% of the sample had at least four comorbidities. The most common comorbidity was diabetes (37.1%), HTN (36.6%), followed by dyslipidemia (29.7%), while the least common condition was depression (3.1%).

During the index hospitalization, COVID-19 patients stayed longer at hospitals (7 vs. 4 days), had a higher transfer to ICU (16.30% vs. 14.34%), and higher death rates (10.48% vs. 5.33%) than pneumonia patients. 

While age did not differ between patients with COVID-19 and pneumonia, the former was slightly increased in females (51.1% vs. 48.8%; *p*-value = 0.02; Table 1). Patients admitted due to COVID-19 were less likely to have any comorbidities, except for dyslipidemia (28.7% vs. 30.2%; *p*-value = 0.11) and depression (3.2% vs. 3.1%; *p*-value = 0.73). 

Overall, the median hospital length of stay (LOS) was five days, but COVID-19 patients were more likely to stay longer (7.0 vs. 4.0; *p*-value < 0.01). COVID-19 patients were more likely to be transferred to the Intensive Care Unit (ICU) than the pneumonia group (16.3% vs. 14.3%; *p*-value < 0.01). At the same time, COVID-19 patients were significantly more likely than pneumonia patients to be readmitted within 30 days (11.1% vs. 8.4%; *p*-value < 0.01; Table 2), with less likely mortality (1.5% vs. 8.0%; *p*-value < 0.01). 

### 3.2. Comparisons between COVID-19 and Pneumonia Patients 

Table 3 depicts the results of comparing COVID-19 to pneumonia patients on various factors. COVID-19 patients were 69% more likely to be admitted into the ICU, and the association remained similar after adjusting for readmission status and mortality. On the other hand, COVID-19 patients have a fewer risk of death than patients with pneumonia. This estimate decreased slightly after adjusting for other covariates (HR = 0.18, 95% CI = 0.14–0.24; Table 3). 

Of the included population, 9.4% were readmitted within 30 days. When we compared reasons for readmission, there were some variations (Table 4). Pneumonia patients were significantly more likely to be readmitted due to pulmonary conditions (57.5% vs. 42.1%; *p*-value < 0.01), while COVID-19 patients were more likely to be admitted due to planned or irrelevant admission (11.2% vs. 0.74%; *p*-value ≤ 0.01). 

COVID-19 patients were more likely to be readmitted, as results from both univariate and multivariate analysis suggest (Table 5). In the univariate analysis, COVID-19 was associated with 37% higher readmission odds than pneumonia. The estimate was even higher (OR = 1.9) when adjusting for age, gender, comorbidities, or whether the patient was admitted to the ICU. There was an increased risk of readmission as the number of comorbidities increased, with those with four comorbidities or more at the highest risk (OR = 2.0). Although diabetes was associated with readmission in the univariate analysis, it was no longer significant in the multivariate analysis. On the other hand, cancer remained a significant predictor of readmission after adjusting for other covariates. 

**Figure 2:**
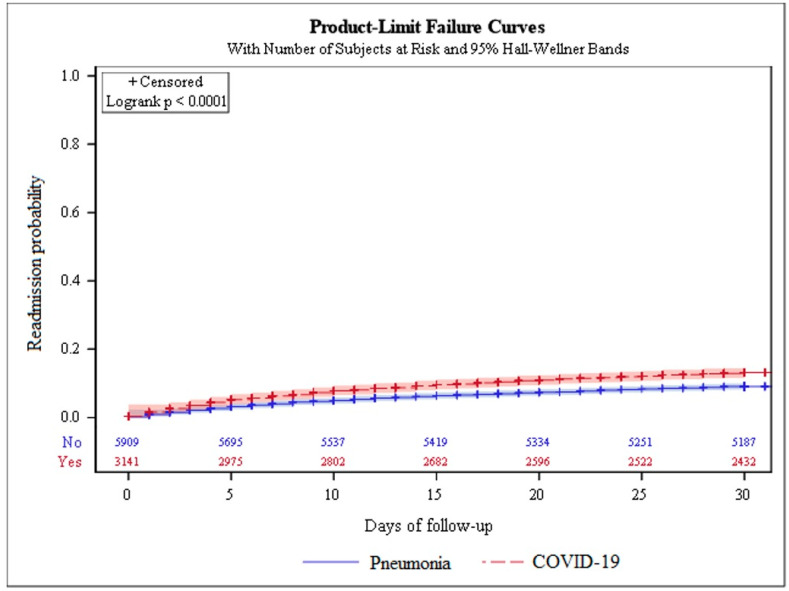
The 30 day risk of hospital readmission between COVID-19 and pneumonia patients.

Figure 2 depicts the risk of 30-day hospital readmission among COVID-19 and pneumonia patients. The findings indicate that there is a statistically significant difference in the chance of readmission. Those with COVID-19 infection had a greater risk of readmission than pneumonia patients (*p*-value ≤ 0.0001).

In additional analyses, we investigated the extrapulmonary reasons for readmission. About 7.23% of the readmitted were with renal diseases, 1% of them with acute kidney injury. In addition, 7% of the readmitted were due to cardiovascular diseases, including 4.19%, 0.34%, and 1.13% that were readmitted due to decompensated heart failure, arrhythmia, and myocardial infarction. Very few patients (0.6%) presented with hyperglycemia. 

## 4. Discussion

The present study examined a large cohort of COVID-19 patients compared to pneumonia patients by assessing readmission to hospitals within 30 days, length of hospital stay, transfer to the ICU, and death. During the index hospitalization, COVID-19 patients stayed longer at hospitals (7 vs. 4 days), had a higher transfer to ICU (16.30% vs. 14.34%), and higher deaths (9.40% vs. 4.60%). After discharge, more COVID-19 patients were readmitted to hospitals (11.15% vs. 8.36%), with higher 30-day death levels (8.23% vs. 7.37%). Although the majority of readmissions were due to pulmonary reasons, especially among pneumonia patients, a sizable portion of COVID-19 patients were readmitted due to planned or uncategorized admission. 

Using the United States Veteran Affair Healthcare system, the 60-day readmission was found to be 20% of the index hospitalization [9]. Though the reasons for readmission were COVID-19, sepsis, pneumonia, and heart failure, the study did not find any significant differences compared to non-readmitted COVID-19 except in age, where there were more older survivors were among the readmitted cases. Our results show differences in reasons for readmission for pulmonary complications that were higher in pneumonia patients. This finding could be due to underlying conditions since the pneumonia patients had significantly higher HTN, asthma, arthritis, cancer, diabetes, and stroke. 

Given that COVID-19 primarily infects the respiratory cells, most readmissions were due to pulmonary disease. Nonetheless, other extrapulmonary reasons for readmission include renal, cardiovascular, and other systems. Despite the small number of readmitted patients with recorded reasons for readmission, COVID-19 patients were more likely to be hyperglycemic or have arrhythmia and/or myocardial infarction. A recent study has investigated differences in hospitalization outcomes between COVID-19 and flu patients and found that COVID-19 patients were more likely to present with these manifestations during index hospitalization [34]. While we have investigated these manifestations during the readmission period, a prior study focused only on the index hospitalization. 

Moreover, we found that death during index hospitalization and within 30-day readmission was more common among COVID-19 patients (see Figure 1 and Table 2). While this finding is comparable to prior studies [34,35], it is unclear why death beyond the 30-day readmission was higher in pneumonia patients. As noted previously, our pneumonia cohort has higher comorbidity than COVID-19 patients, which could contribute to their subsequent deaths during longer readmission periods. Given the scope of our research, mainly readmission and death during 30 days, the results are unlikely to be impacted by events beyond 30 days.

In our study, LOS was longer for COVID-19 patients, but this could be due to extra precautions practiced by healthcare systems, especially in early 2020 when there was scarce information on COVID-19 at the time. Moreover, the strong positive association between LOS and hospital readmission in our cohort could explain the higher readmission rate among COVID-19 patients than among pneumonia patients. Overall death was more prevalent in the pneumonia patients, but this can be a result of complicated cases, since 87.56% of those patients had at least four comorbidities, compared to 12.45% of COVID-19 patients. 

In sensitivity analyses, we assessed the risk of 30-day hospital readmission after removing readmission due to planned/irrelevant admission (see Table 2, Table 3 and Table 5). Compared to patients discharged from the hospital because of pneumonia, those who were readmitted due to COVID-19 had similar results as in the original analysis. This finding reinforces the higher readmission in COVID-19 patients despite the higher planned/irrelevant readmission. Additionally, while mortality among COVID-19 patients was higher compared to the readmission period, the rates during the readmission period were stable. On the contrary, the mortality rate during index hospitalization in pneumonia patients was lower than those during the readmission period. 

One of the strengths of the present study is the use of data from the largest healthcare systems in Saudi Arabia, which covers different regions of the country. Eligible members have equal access to care within this integrated system of similar diagnostic and treatment plans, facilitating readmission comparisons. Despite the strengths, several limitations should be considered when interpreting our findings. First, we only considered the index hospitalizations for the COVID-19 and pneumonia patients, and any further readmissions were not counted. Second, similar to other electronic health records, imprecise measurements, potential misclassifications, and residual confounding is possible. Nonetheless, we augmented our diagnoses with available lab results to minimize the potential biases. 

## 5. Conclusions

Patients who had recovered from COVID-19 encountered more frequent hospital readmissions than pneumonia patients with respiratory and non-respiratory presentations. The findings of this cohort analysis will inform the current discussion about readmission and death in COVID-19 patients. 

## Figures and Tables

**Table 1:** Baseline demographic and clinical characteristics of patients admitted to hospital due to COVID-19 or pneumonia, NGHA, Saudi Arabia.

Characteristics *n* (%)	All (*n* = 9921)	COVID-19 (*n* = 3597)	Pneumonia (*n* = 6324)	*p* Value
Age, mean (SD)	52.00 (26.79)	52.27 (18.15)	51.85 (30.65)	0.38
Gender				
Male	5002 (50.42)	1759 (48.90)	3243 (51.28)	0.022
Female	4919 (49.58)	1838 (51.10)	3081 (48.72)	
Comorbidities				
HTN	3630 (36.59)	1084 (30.14)	2546 (40.26)	<0.01
Asthma	1027 (10.35)	142 (3.95)	885 (13.99)	<0.01
Arthritis	242 (2.44)	43 (1.20)	199 (3.15)	<0.01
Cancer	765 (7.71)	175 (4.87)	590 (9.33)	<0.01
Depression	312 (3.14)	116 (3.22)	196 (3.10)	0.73
Diabetes	3679 (37.08)	1238 (34.42)	2441 (38.60)	<0.01
Dyslipidemia	2946 (29.69)	1034 (28.75)	1912 (30.23)	0.11
Stroke	975 (9.83)	176 (4.89)	799 (12.63)	<0.01
Number of comorbid conditions				
None	2067 (32.69)	1670 (46.43)	2067 (32.69)	<0.01
1	2085 (21.02)	683 (18.99)	1402 (22.17)	
2	1236 (12.46)	414 (11.51)	822 (13.00)	
3	1513 (15.25)	495 (13.76)	1018 (16.10)	
≥4	1350 (13.61)	335 (9.31)	1015 (16.05)	

HTN: Hypertension.

**Table 2:** Outcomes of patients discharged from the hospital with COVID-19 or pneumonia, NGHA, Saudi Arabia.

Outcome, *n* (%)	All (*n* = 9921)	COVID-19 (*n* = 3597)	Pneumonia (*n* = 6324)	*p* Value
LOS, median (Inter quartile range	5 (7)	7 (7)	4 (7)	<0.01
Transfer to ICU	1493 (15.05)	586 (16.29)	907 (14.34)	0.0073
30-day readmission	930 (9.37)	401 (11.15)	529 (8.36)	<0.01
Overall death	563 (5.67)	55 (1.53)	508 (8.03)	<0.01
Death within 30-day	(33/401) = 8.23%	(33/401) = 8.23%	39/529 = 7.37%	

LOS: Length of stay; ICU: Intensive Care Unit.

**Table 3:** Risk of hospital readmission and deaths in patients admitted to hospital with COVID-19 compared to pneumonia patients, NGHA, Saudi Arabia.

Outcomes	Crude Hazard Ratio (95% CI)	Adjusted Hazard Ratio (95% CI)
Transfer to ICU	1.80 (1.62, 2.01)	1.85 (1.65, 2.07)
Readmission	1.49 (1.31, 1.69)	1.68 (1.46, 1.92)
Death	0.15 (0.11, 0.19)	0.18 (0.14, 0.24)

**Table 4:** Reasons for 30-day hospital readmission among discharged patients with COVID-19 and pneumonia, NGHA, Saudi Arabia.

	COVID-19 (*n* = 401)		Pneumonia (*n* = 543)		*p* Value
	N	%	N	%	
Pulmonary	169	42.14	312	57.46	<0.01
Hematologic	19	4.74	27	4.97	0.86
Cardiovascular	24	5.99	38	7.00	0.53
Neuropsychiatric	6	1.50	9	1.66	0.85
Renal	29	7.23	30	5.52	0.28
Endocrine	7	1.75	6	1.10	0.42
GI and hepatobiliary	41	10.22	41	7.55	0.15
Musculoskeletal	6	1.50	5	0.92	0.60
Ear, nose and throat	0	0	2	0.37	0.66
Dermatology	0	0	4	0.74	0.21
Irrelevant/planned admission	45	11.22	4	0.74	<0.01
Missing	55	13.72	65	11.97	0.42

**Table 5:** Univariate and multivariate analysis for 30-day hospital readmission among patients discharged from hospital with COVID-19 and pneumonia, NGHA, Saudi Arabia.

Clinical Manifestations	Readmitted (%)	None-Readmitted (%)	Crude Odds Ratio (95% CI)	Adjusted Odds Ratio (95% CI)
**Index hospitalization**				
Pneumonia	529 (8.36)	5795 (91.64)	Reference	Reference
COVID-19	401 (11.14)	3196 (88.85)	1.37 (1.19, 1.57)	1.90 (1.61, 2.24)
**Age**				
≤10	87 (6.49)	1254 (93.51)	1.72 (0.97, 3.07)	2.02 (1.13, 3.62)
11–20	14 (3.87)	348 (95.13)	Reference	Reference
21–30	61 (8.37)	668 (91.63)	2.27 (1.25, 4.12)	1.85 (1.01, 3.38)
31–40	60 (7.24)	769 (92.76)	1.94 (1.06, 3.52)	1.40 (0.76, 2.56)
41–50	59 (7.60)	717 (92.40)	2.04 (1.12, 3.71)	1.35 (0.73, 2.47)
51–60	98 (8.11)	1111 (91.89)	2.19 (1.24, 3.88)	1.34 (0.74, 2.40)
61–70	157 (9.46)	1502 (90.54)	2.59 (1.49, 4.54)	1.66 (0.93, 2.94)
71–80	199 (12.20)	1432 (87.80)	3.45 (1.98, 6.09)	2.34 (1.32, 4.14)
81–90	163 (14.78)	940 (85.22)	4.31 (2.46, 7.54)	3.01 (1.70, 5.37)
≥91	32 (11.35)	250 (88.65)	3.18 (1.66, 6.09)	2.46 (1.27, 4.76)
**Gender**				
Male	483 (9.66)	4519 (90.34)	Reference	Reference
Female	447 (9.09)	4472 (90.91)	0.93 (0.82, 1.07)	
**LOS (median, IQR)**	6.0 (10.0)	5.0 (7.0)	1.004 (1.001, 1.006)	
**Comorbidities**				
**HTN**				
Yes	419 (11.54)	3211 (88.46)	1.48 (1.28, 1.69)	
No	511 (8.12)	5780 (91.88)	Reference	Reference
**Asthma**				
Yes	94 (9.15)	933 (90.85)	0.97 (0.78, 1.21)	
No	836 (9.40)	8058 (90.60)	Reference	Reference
**Arthritis**				
Yes	35 (14.64)	204 (85.35)	1.00 (0.83, 1.20)	
No	900 (9.29)	8783 (90.70)	Reference	Reference
**Cancer**				
Yes	137 (17.91)	628 (82.09)	2.30 (1.89, 2.81)	2.18 (1.77, 2.70)
No	793 (8.66)	8363 (91.34)	Reference	Reference
**Depression**				
Yes	38 (12.18)	274 (87.82)	1.35 (0.96, 1.92)	
No	892 (9.28)	8717 (90.71)	Reference	Reference
**Diabetes**				
Yes	400 (10.87)	3279 (89.13)	1.31 (1.15, 1.51)	
No	530 (8.49)	5712 (91.51)	Reference	Reference
**Dyslipidemia**				
Yes	332 (11.27)	2614 (88.73)	1.35 (1.17, 1.56)	
No	598 (8.57)	6377 (91.43)	Reference	Reference
**Stroke**				
Yes	154 (15.79)	821 (84.21)	1.97 (1.64, 2.38)	1.66 (1.34, 2.05)
No	776 (8.67)	8170 (91.33)	Reference	Reference
**No. of comorbidity**				
None	240 (6.42)	3497 (93.58)	Reference	Reference
1	222 (10.65)	1863 (89.35)	1.74 (1.43, 2.10)	1.53 (1.25, 1.88)
2	129 (10.44)	1107 (89.56)	1.69 (1.36, 2.13)	1.39 (1.08, 1.79)
3	172 (11.37)	1341 (88.63)	1.86 (1.52, 2.29)	1.47 (1.16, 1.87)
≥4	167 (12.37)	1183 (87.63)	2.06 (1.67, 2.53)	1.36 (1.05, 1.77)
**Transfer to ICU**				
yes	176 (11.79)	1317 (88.21)	1.36 (1.14, 1.62)	1.30 (1.09, 1.56)
No	754 (8.95)	7674 (91.05)	Reference	Reference

HTN: Hypertension.

## Data Availability

The data are available from the PHRS, but restrictions apply to the availability of these data due to sensitive identifiers used in this study, which were used under license for the current study and so are not publicly available.
